# Physician-Delivered Pain Neuroscience Education for Opioid Tapering: A Case Report

**DOI:** 10.3390/ijerph17093324

**Published:** 2020-05-11

**Authors:** Vikas Agarwal, Adriaan Louw, Emilio J. Puentedura

**Affiliations:** 1 Department of Internal Medicine, Mosaic Life Care, St. Joseph, MO 64506, USA; vikasagar@gmail.com; 2 Pain Science Division, Evidence in Motion, San Antonio, TX 78247, USA; adriaan@eimpt.com; 3 Robbins College of Health and Human Sciences, Baylor University, Waco, TX 76798, USA

**Keywords:** chronic pain, pain neuroscience education, opioid, opioid epidemic, opioid tapering

## Abstract

We describe the case of a 75-year-old female with chronic low back pain (CLBP), on opioids for more than 15 years. She presented with an acute episode of nausea, vomiting, abdominal pain, and shortness of breath. After a complete work-up, it was concluded that her presenting symptoms were likely due to her high levels of CLBP and high dose opioids. At the time of intervention, her opioid dosage was between 50–90 MME (Morphine milligram equivalent) (Norco 8 × 7.5 mg/day + Fentanyl 12 mcg patch). She was subsequently seen by the physician for seven outpatient internal medicine appointments over nine months and received Pain Neuroscience Education (PNE) in conjunction with monitored tapering of opioids and other medication associated with her CLBP. This case report demonstrates how a physician might deliver PNE as a viable nonpharmacological treatment option for the tapering of long-term opioids for chronic pain.

## 1. Introduction

Chronic pain is estimated to affect somewhere between 11% to 40% of the United States (US) population, i.e., approximately 36.5 to 132 million Americans [1,2]. It is also reported to affect approximately 20% of the European population (about 150 million) and it is more prevalent in women, older people, and those of lower socio-economic status [3]. The management of chronic pain has become a problem which not only affects medical practice, as it reduces working ability and affects society in general [4]. In the US, heavy reliance on the pharmacologic management of chronic pain has led to an opioid use epidemic, which is characterized by two interrelated phenomena. These are the abuse of opioids that are commonly prescribed as pain medications, and their substitution with illicit opioids such as heroin [5]. Hydrocodone (e.g., Vicodin, Lortab) is the most prescribed opioid in the USA and the 2017 annual prescribing rate per 100 persons was 58.5 for all opioids [6], and it is also the most often abused. It is now well understood that continuing opioids for chronic pain is associated with opioid-induced-hyperalgesia, which increases the patient’s pain experience, and the development of opioid tolerance, which increases the risk of addiction and might well fuel the opioid epidemic [7,8,9]. It is within this context that current best-practice guidelines call for greater utilization of multidisciplinary, non-pharmacologic treatments in the management of chronic pain [10,11].

Opioid misuse, abuse, and addiction in the management of chronic pain is well documented [12]. It has been shown that 13.5% of patients who take opioids for eight days will still be taking them one year later, and 30% of patients who take opioids for 31 days will still be using them one year later [8]. The tapering of opioids is complicated and rooted in various issues pertaining to addiction [9,13,14]. Ironically, tapering from opioids involves an alternative opioid medication, and often, the patient’s current opioid medication is used for the taper [15]. Although evidence for the most effective tapering strategy is lacking [16], the objective is to reduce the opioid dose at a rate that does not produce withdrawal symptoms and pain flare ups [16,17,18]. Endogenous opioids, such as endorphins and enkephalins, are thought to be downregulated in chronic pain states [19,20], and especially so in patients with high pain catastrophizing [21]. Studies on the neurobiological underpinnings of placebo and nocebo effects have shown that higher levels of pain catastrophizing have been linked to lower levels of endogenous opioids and cannabinoids (endocannabinoids) [22,23]. Conversely, decreased levels of pain catastrophizing have been linked to increased levels of these naturally substances [22,23]. Furthermore, emerging research has shown that conservative treatments, such as a combination of exercise (with pacing and graded exposure) and cognitive training (education), can result in increased release and restoration of these endogenous opioids [24,25,26].

Pain neuroscience education (PNE) is an emerging cognitive training program that physicians may consider providing to their patients with chronic pain [11,27]. The aim of PNE is to teach patients more about the biology and physiology of their pain experience [28]. Various systematic reviews and meta-analyses have proven its efficacy in positively influencing pain, disability, fear-avoidance, and pain catastrophizing [29,30,31]. PNE, which originated from within the physical therapy profession, has since been successfully taught to other professions, including physicians and physician assistants [27,32]. Recent research into PNE has shown that shorter, clinically meaningful sessions result in significant changes in pain catastrophizing which should be a key target in reengaging the endogenous mechanisms [24,30]. It is postulated that PNE might be ideal for today’s busy physician who might wish to provide a safer, evidence-based approach to tapering a patient with persistent pain using opioids because of its efficacy in directly targeting pain catastrophizing and because it can be delivered in relatively short durations. This case study demonstrates the delivery of PNE by an internal medicine physician to a patient with chronic pain and opioid use.

## 2. Case Report

Permission to report this case was obtained from the patient, who was a 75-year-old female reporting chronic low back pain (CLBP) for more than 20 years. She had seen an Interventional Pain Specialist and undergone several series of epidural injections, with the first in 2006 and her last injections in 2016. She reported taking opioids for more than 15 years and would see her physician four times a year. She had been diagnosed with CLBP secondary to degenerative disc disease, acquired scoliosis, and lumbosacral spondylosis.

Past medical history included rheumatoid arthritis affecting her hands, wrists, and ankles, and she was seeing a Rheumatologist who had her taking Leflunomide, a disease modifying anti-rheumatic drug (DMARD) for the past five years. Other relevant past history included fibromyalgia, for which she was taking Nortriptyline; bilateral total knee replacements and right distal femur fracture for which she had open reduction internal fixation; obesity (her BMI was 36); gastroesophageal reflux disease (GERD); mild coronary artery disease for which she had undergone cardiac catheterization twice within the past 10 years; and, recurrent falls.

The patient was a widow, non-smoker, who lived by herself. She had retired from her work as the director of a day care center and she used a cane as an aid for ambulation. She reported she had low functional status.

Seven months prior to her initial physician visit, she developed abdominal pain (epigastric region) with prolonged nausea and constipation. Medical work-up, including imaging and blood tests, was unremarkable. It was suggested to her that her long-term use of opioids might have contributed to some of her acute abdominal symptoms.

One month before her initial physician visit, she attended the emergency department with acute onset of chest pain (Numeric Pain Rating Scale [NPRS]—9/10), nausea, vomiting, abdominal pain and shortness of breath. For the chest pain, cardiology diagnosed and successfully treated for non-ST-elevated myocardial infarction. Her abdominal ultrasound was unremarkable, and the same internal medicine physician saw the patient for epigastric discomfort and nausea. The attending physician was of the opinion that her abdominal pain and nausea was likely due to her high levels of CLBP (NPRS—7/10) and high dose of opioids (Norco 8 × 7.5/325 milligram (mg)/day and Fentanyl 12 mcg patch every 72 h). She was educated regarding the side-effects of high-dose opioids, opioid-induced-hyperalgesia, and the need to taper opioids. She was receptive and was scheduled for subsequent outpatient follow-up at internal medicine one week later.

## 3. Visit 1

At the time she presented as an outpatient, her CLBP was rated as 7/10. She reported her current pain across her lower back without radiation into the lower extremities, which would increase with standing and walking, ease with rest, medications, and use of heat. She reported taking 8 Norco 7. 5/325 mg per day orally; Pamelor (Nortriptyline) 10 mg at night; Voltaren (Diclofenac) 1% gel 2 gm topical for times per day; and transdermal Fentanyl patch 12 mcg/hr extended release every 72 hours. Her nausea had improved, but she complained of generalized fatigue.

As part of her outpatient screen, the patient completed an adapted, internal 13-question Yellow Flags Questionnaire (YFQ) to ascertain potential biopsychosocial risk factors [33]. The YFQ assesses four domains, including pain (Question 1), health confidence (Questions 3, 4, 6 and 8), fear-avoidance (Questions 9, 11, 12, and 13) and emotional (Questions 5, 7, and 10). Although it has not been validated, the yellow-flags screening tool was designed to allow for a quick screen across the various domains by a physician and is based on previously validated questionnaires, such as the fear-avoidance beliefs questionnaire, pain catastrophizing scale, etc. Table 1 shows her initial YFQ scores (total 65/130) and her final visit YFQ scores (total 26/130) seven months later (in various aspects (pain Q1, insomnia Q10, etc.). The patient scored high on being significantly impacted by her CLBP. These high scores on psychosocial issues, along with disproportionate pain, disproportionate aggravating and easing factors, and diffuse palpation tenderness, were consistent with the clinical presentation of a nociplastic dominant (central sensitization) clinical presentation [34]. Cognitive Behavioral Therapy (CBT) and PNE have both shown to be a first-choice treatment for patients presenting with a nociplastic pain presentation [11,30]. The attending internal medicine physician recently participated in a PNE continuing medical education class and decided to utilize PNE, especially since it could be done in abbreviated sessions and it had shown efficacy in easing pain and improving movement [28,30]. PNE utilizes metaphors, examples and images to teach patients more about the neurobiology and neurophysiology of their pain experience [30,35,36]. In all, the patient attended seven outpatient internal medicine appointments over a nine-month period (Table 2). Typical sessions included review of systems, consultation with the physician, specific PNE-targeted sessions and specific home exercise programs (HEP). The duration of each session was between 25 and 45 min and the format included verbal discussion, YouTube videos, Why You Hurt card system [37], and other patient education sheets showing a compilation of important figures and concepts from Explain Pain Supercharged [36]. Patient educational material was given at each session for her to reflect upon and bring back to subsequent sessions, and the patient also kept a file of her PNE sessions, which she could frequently access and further discuss at each visit. Table 1 lists details of each visit.

## 4. Visits 2–7

**Figure 1:**
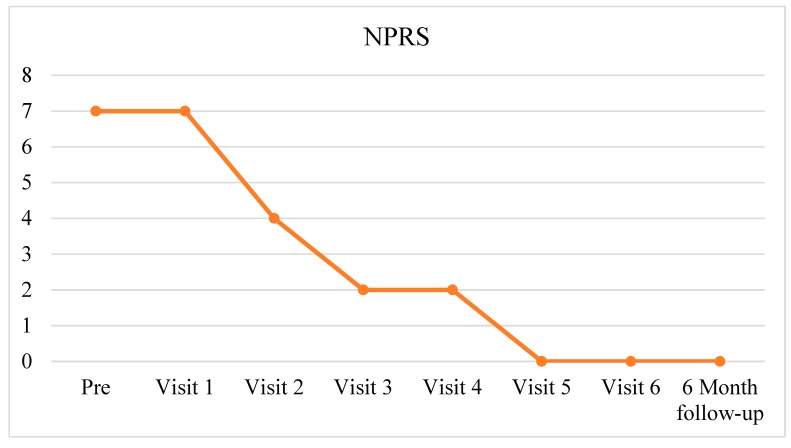
Self-reported low back pain rating (NPRS).

**Figure 2:**
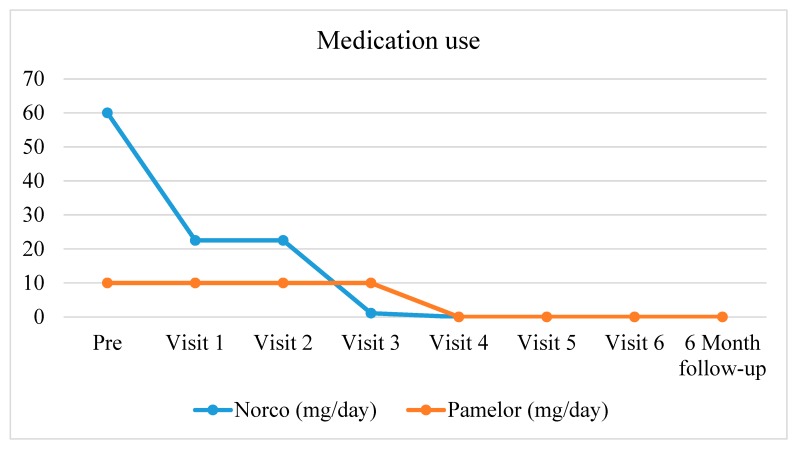
Medication-use for CLBP over the course of the treatment (milligrams/day).

The attending physician tapered opioids and other medicine associated with her CLBP, depending on her responses. Over the 12-week period (89 days since discharge from the hospital), the patient’s CLBP decreased from 7/10 to 0/10 (Figure 1) and opioids and antidepressants were completely abolished (Figure 2). Both of these positive results were still intact at the final six-month follow-up.

## 5. Discussion

A recent systematic review involving qualitative evidence synthesis using meta-ethnography reported that people taking opioids for chronic non-cancer pain were constantly balancing tensions and not always wanting to take opioids [38]. Many weighed the pros and cons of taking opioids but felt that they had no choice because of their pain [38]. They frequently felt stigmatized and not always ‘on the same page’, as their prescribing physician and felt changes in opioid use were often very challenging [38]. Opioid stewardship has become a major concern among prescribing physicians, and there are a growing number of guidelines and protocols for tapering opioids [39,40,41,42]. However, the misapplication of 2016 opioid prescribing guidelines from the US Centers for Disease Control and Prevention (CDC) [16] has been cited as a significant concern and contributing factor for abrupt tapering or discontinuing opioids in patients who have been stable on such medication for years [43]. This has led to an international stakeholder community of pain experts to call for urgent action on forced opioid tapering [44], and the publication of the EMPOWER study protocol that aims to provide evidence on patient response to voluntary opioid tapering [45]. The EMPOWER study will involve three study arms (1) group cognitive behavioral therapy; (2) group chronic pain self-management; and, (3) usual care (taper only) [45].

To our knowledge, there are no studies or case reports involving physicians using PNE in their clinical practice as an adjunct to tapering of opioid medications. We did find one commentary on a case scenario, which promoted a BRAVO (broaching the subject, risk-benefit calculation, addiction, velocity and validation, other strategies) protocol [41], which was based on expert consensus and emerging evidence and it was thought to provide a safe and compassionate framework for opioid tapering [41]. Our case report illustrates the process in which a physician utilized PNE in clinical practice and it was able to taper a patient off opioids for their CLBP. While the content and delivery method were not substantially different to similar case reports [46,47,48,49], in this case report the clinician providing the PNE was a physician that was trained in the approach.

Although modern healthcare is thought to be delivered by multidisciplinary, distributed healthcare teams, there is often a lack of teamwork and communication, which can lead to ineffective and unsafe patient care [50,51]. This may be most evident in the management of chronic pain. In this case report, our patient visited numerous healthcare providers over the course of her CLBP and various other comorbidities. Each of these healthcare interactions were potential opportunities to address her long-term opioid use and, yet, it was not addressed until she presented at the emergency department with acute chest pain. Physicians have significant power when it comes to influencing patients, both positively and negatively [52,53]. The prescription of opioid medication to address chronic pain without appropriate education regarding the consequences of long term use might be an example of a negative influence. This case report illustrates a physician providing a positive influence, despite it being after a 10-year episode of CLBP. One wonders what the outcome might have been for this patient if a physician, well-versed in a biopsychosocial approach, had provided her with such an approach earlier in her path.

Some of the latest research into PNE has focused on the requirements for a meaningful interaction between the patient and clinician. The trust between the patient and clinician is one of the biggest predictors of success with that interaction [54]. This is felt to be relevant, because, in patients with chronic pain, such as in this case report, nociceptive pathways of the pain neuromatrix can shift over time to the more emotional circuitry, ultimately targeting the amygdala [55]. It has been shown that with increased activation of the emotional circuitry, and specifically the amygdala, patients will display more untrustworthy judgments towards their healthcare providers [56]. This, in turn, can fuel pain catastrophizing, which has been linked to endogenous anti-opioid and anti-cannabinoid expression, which then increases pain [23]. Physicians might well wonder how they might build enough time into their busy practice to spend on developing trust and teaching their patients more about their pain (PNE). The good news (clinically) is that trust has poor correlation to length of time spent with a patient and, thus, can be established in a short amount of time by an attentive, present physician. This is important, as it is based on the premise that physicians with each contact have the ability to powerfully influence a patient’s behavior, including the taking of opioids for pain relief. While the multidisciplinary model of healthcare would have the patient referred to other healthcare providers to address many of the underlying issues in greater depth, it will not work without the appropriate teamwork and communication. Until such teamwork and communication is in place, the physician-patient interaction should be viewed as a powerful, frontline approach in the management of chronic pain.

One of the biggest challenges in managing patients with chronic pain is the lack of consistency in the message that patients are given about their pain [57]. Healthcare providers may not speak the same language and they often provide very differing and, therefore, confusing explanations for why a patient may be experiencing chronic pain. Proponents of PNE contend that patients need to be presented with scientific and biological explanations, so that they can clearly understand the ‘why’ and ‘how’ of their continued pain experience [30]. Because many of these patients have been managed with a pathoanatomical model of pain, they need a lot of ‘de-education’ to reconceptualize their pain [58]; otherwise, there are significant impediments to their embracing the biopsychosocial nature of pain.

Case reports have significant limitations due to their design and are considered to be the lowest level of evidence, but they are often the first line of evidence, because they may present new ideas for research. They may also serve to remind clinicians of powerful tenants underscoring daily clinical practice. We hope that this case report might be seen as doing both. First, it might suggest or encourage larger studies exploring physician-delivered PNE and the use of PNE-based treatments to taper opioid use. Second, it illustrates how a caring physician noticed that their patient was in desperate need of help and it was able to provide hope, which has been shown to enhance naturally occurring endogenous mechanisms [23]. This case study should remind all healthcare providers of the speech made by Dr. Rudyard Kipling nearly 100 years ago in 1923 to the Royal College of Surgeons in London: “Words are, of course, the most powerful drug used by mankind.”

## 6. Conclusions

We consider that physician delivered PNE was an important factor in the successful management and tapering off opioids in a patient with CLBP. This case report suggests that physicians can deliver PNE as a viable nonpharmacological treatment option for the tapering of long-term opioids for chronic pain. Controlled studies are needed in order to confirm the benefit of this approach in clinical practice.

## Figures and Tables

**Table 1:** Pre-intervention and post-intervention Yellow Flags Questionnaire (YFQ) scores.

	Pain (Q 1)	Health Confidence (Qs 3, 4, 6 and 8)	Fear-Avoidance (Qs 9, 11, 12 and 13)	Emotional (Qs 5, 7 and 10)	Total Score (All 13 Qs) *
	(0–10)	(0–40)	(0–40)	(0–30)	(0–130)
Initial Visit—	7	21	18	15	65
Final Visit—9 months	3	8	10	5	26

* Q 2 is not included in the 4 domains but is added to the total score.

**Table 2:** Evaluation findings and interventions provided.

Visit	Information	Treatment
1	Follow-up visit 5 days after discharge from hospital Nausea improved Willing to talk about her CLBP and opioid use “What do you think is going on with your back?” “I had a scan and it’s full of arthritis” Currently taking 2-3 Norco for her LBP Could not handle Fentanyl patches Lots of fatigue Decision: McKenzie/ Mechanical intervention PNE Patient Reported Outcome measures: Yellow Flag Risk Form 65/130 Pain 7/10 Anxiety 3/10 Depression 2/10 Insomnia 8/10	Education about the normal anatomical changes and aging of her low back Disconnecting the belief that aging and pain are synonymous Current normative data on imaging and pain ○ “Arthritis” ○ Bulging discs ○ Degenerative Disc Disease (DDD) PNE: Central sensitization explained as a “sensitive alarm system” How psychosocial yellow flags result in a “sensitive alarm system” Cognitive homework: Identify and review her personal yellow flags Explore how a “sensitive alarm system” has limited her movement and function versus the health of her tissues Thresholds for activities before and after pain
2 (5 days later)	Follow-up visit LBP 4/10 Completed her sensitive nerves and yellow flags homework Her questions at follow-up: “Could this have anything to do with my RA or fibromyalgia?” “What kind of medicines does my brain make to help me feel better?” “Does weather have anything to do with pain?” “How come I can go 3 or 4 days with only one Tylenol and maybe one Norco, then suddenly be in pain, and have to take up to 3 Norco and still be in pain?” The activities she listed before and since developing pain (used-to-could): Shop for hours Do a lot of walking Gardening Climbing ladders – wash windows and change lightbulbs Refinish furniture Yellow flags/keeping the alarm system elevated: Overdoing chores Upset and worried Exercise	Review content from session 1 and HEP Discussion of list of opioid side effects and identified those she was suffering from PNE for weather changes and pain: How “nerve sensors” make you aware of weather changes and stress and how it may increase pain Video: Understanding chronic pain (YouTube^TM^) Cognitive homework: Review education from this and previous session Reflect on content Bring back any questions Make a list of and reflect on opioid side-effects
3 (9 days later)	Review side-effects of opioids reflection Over the last 3 weeks she has only taken 3 Norco and has done fairly well Less constipated Dry mouth is improving Pain is improved – which surprises her LBP 2/10 Encourage her to stop Norco	Review content from session 2 and HEP PNE: Explain Pain Supercharged nuggets — Normalizing the pain experience and “not freaking out” — It won’t happen overnight, but it will happen. Realistic goals and the journey of pain — We grow like trees and reflect her story over time; normalizing scan findings and sensitization of the nervous system over time PNE: Stress responses to pain via lion metaphor Cognitive Homework: — Identify and record daily stress responses
4 (7 days later)	Follow-up Done her homework States she is “better than before”—pain and outlook towards life is improved Not been taking any Norco Food tastes better Dry mouth continues to improve Does not feel as “groggy” as before—“more in tune with people” around her Realizing she was staying more at home—deliberately trying to go out more LBP 2/10 Discontinued Colace since she was no longer constipated Pamelor (nortriptyline) 10mg (anti-depressant) nightly discontinued See her again in 2 weeks	Review content from session 3 and HEP Additional education—myths about LBP: Myth 1: Pain is inevitable with aging Myth 2: If you have some pain now, then you will have worse pain later Myth 3: Toughing it out makes it easier to tolerate Myth 4: There is nothing you can do about it PNE: Review metaphor of “kisses of time and growing like trees” Complexity of pain—“traffic jams, cakes and snowflakes”
5 (14 days later)	Follow-up Reports no pain “Frustration and stress make me uncomfortable in my back”—does not use the “P” word anymore Reports no problem with discharging her Pamelor No constipation anymore She discontinued her Prilosec since she’s not having any heartburn anymore	Review content from sessions 1-4 Reflect on PNE and application to her specific presentation of CLBP Questions/answers
6 (7 weeks later)	Follow-up Off Norco for a few months No pain—feels much better Much more active More energy Had a slight flare recently but did not freak out and no medicine, including over the counter medicine No heartburn; no abdominal pain Improved mood; not anxious or depressed; no sleep disturbance LBP 0/10 Patient brought all her prescription and OTC medicines to the clinic—review all medicines and update her current medication list in the medical records to reflect the changes Follow-up in 6 months	Review content from sessions 1-5 Reflect on PNE and application to her specific presentation of CLBP Questions/answers
7 (6 months later)	Follow-up Continued all opioid medications Little to no pain experienced Still active and feeling energetic Patient Reported Outcome measures: Yellow Flag Risk Form 26/130 (30% reduction)	
